# A Reference Hand-Book of the Medical Sciences

**Published:** 1889-04

**Authors:** 


**A Reference Hand-Book of the Medical Sciences, embracing the Entire Range of Scientific and Practical Medical Science.** By various writers. Illustrated by chromo-lithographs and fine wood engravings. Edited by ALBERT H. BUCK, M. D. Volume vii. New York: William Wood & Co., 56 and 58 Lafayette place. 1889.

The previous volumes of this admirable work have been favorably noticed in this journal, its scope and object commented upon and the enterprise of the publisher in furnishing to the profession so complete an encyclopedia of medical knowledge, warmly approved. The present volume carries the work from

Teratology to the concluding article on *Worms*, and comprises an excellent selection of subjects from the best writers in this country and also in Canada.

Many of the subjects treated in this work are quite exhaustive, and shows a purpose, upon the part of the writer, to conduct within a small compass the main principles and also the latest thoughts. We may refer to the article of the Tympanic Membranes, which is well illustrated, and shows the anatomy of the ear, the physiology of hearing, and, finally, the pathological conditions which follow from injuries and from disease. The treatment of morbid conditions of the tympanum is also considered. The article on Vaccination, from the pen of Dr. Samuel W. Abbott, Secretary of the State Board of Health of Massachusetts, is a valuable one, and gives the latest opinion of sanitarians in regard to the important measure in the prevention of a dread disease. We have not space to review other and equally valuable articles in this work. The seven volumes, in which the present volume is included, constitute a library in itself. Especially valuable to those who have not access to medical libraries in our large cities, while to the busy practitioner, who has not the leisure to wade through exhaustive treatises, this Reference Hand-Book furnishes just the literature needed, in an able and satisfactory manner.

